# Vergleich von Diagnosedaten nach der Einführung des Mukoviszidosescreenings bei Neugeborenen in Deutschland

**DOI:** 10.1007/s00103-023-03778-1

**Published:** 2023-10-13

**Authors:** Lutz Nährlich, Inken Brockow

**Affiliations:** 1 Abteilung für Allgemeine Pädiatrie und Neonatologie, Justus-Liebig-Universität Gießen, Feulgenstr. 12, 35392 Gießen, Deutschland; 2 Deutsches Mukoviszidoseregister, Mukoviszidose e. V., Bonn, Deutschland; 3 Bayerisches Landesamt für Gesundheit und Lebensmittelsicherheit (LGL), Oberschleißheim, Deutschland; 4 Deutsche Gesellschaft für Neugeborenenscreening e. V. (DGNS), Leipzig, Deutschland

**Keywords:** Mukoviszidose, Zystische Fibrose, Diagnose, Neugeborenenscreening, Falsch-negatives Ergebnis, Cystic fibrosis, Newborn screening, Diagnosis, Missed case, Inconclusive diagnosis

## Abstract

**Hintergrund:**

Zum 01.09.2016 wurde das Neugeborenenscreening (NGS) auf Mukoviszidose (Cystic Fibrosis – CF) in Deutschland eingeführt. Bisher gibt es keinen epidemiologischen Goldstandard für die Erfassung der Diagnosezahlen. Daher werden Daten der in der Konfirmationsdiagnostik bestätigten Fälle der Deutschen Gesellschaft für Neugeborenenscreening (DGNS) und die Diagnosehäufigkeiten des Deutschen Mukoviszidoseregisters (DMR) gegenübergestellt. Dies kann auch die Evaluation des CF-Screenings unterstützen. Ziel der Arbeit ist es, die Daten der DGNS und des DMR zu vergleichen und Limitationen sowie Stärken dieser Datenquellen darzustellen.

**Methoden:**

Analysiert werden die Mukoviszidose-Diagnosedaten der DGNS (Datenstand 14.04.2023) und des DMR (Datenstand 12.04.2023) für 2017–2021 geborene Kinder im Hinblick auf Häufigkeiten, Anzahl falsch-negativ Gescreenter und Verhältnis CF zu „Fällen mit positivem Mukoviszidosescreening und unklarer Konfirmationsdiagnostik“ (CFSPID).

**Ergebnisse:**

Der DGNS liegen 767 Datensätze von Neugeborenen mit CF/CFSPID vor, dem DMR 910 bestätigte Diagnosefälle CF/CFSPID. Ein falsch-negatives Screening wird von der DGNS für 37/767 (4,8 %) und vom DMR für 49/910 (5,4 %) berichtet. Das Verhältnis von CF zu CFSPID beträgt 17,4:1 (DGNS, 2017–2020) bzw. 28,1:1 (DMR).

**Diskussion:**

Die DGNS und das DMR liefern bei unterschiedlichen Stärken in der Dokumentation der Screening-(DGNS) und Diagnosedaten (DMR) wichtige Anhaltspunkte für die Anzahl neu diagnostizierter Mukoviszidosepatienten nach Einführung des NGS. Gesetzliche Vorgaben zur Nachverfolgung der Gescreenten und Erfassung aller Kinder mit Mukoviszidose sowie der Datenaustausch zwischen DGNS und DMR könnten zukünftig die Evaluation verbessern.

## Abstract (en)

**Background:**

In Germany, newborn screening (NGS) for cystic fibrosis (CF) was introduced on 1 September 2016. There is no legally required follow-up of abnormal screening findings, so the exact number of final diagnoses is not known. Two data sources can support the evaluation of the cystic fibrosis screening: the German Society for Newborn Screening (DGNS) collects the results of NGS and confirmatory testing for quality assurance and the German Cystic Fibrosis Registry (DMR) collects diagnostic frequencies. The aim of this manuscript is to compare the data from the DGNS and the DMR and to present limitations and strengths of each data source.

**Methods:**

Data from the DGNS (data as of 14 April 2023) and the DMR (data as of 12 April 2023) for children born between 2017 and 2021 were analyzed with regard to the frequencies of CF, number of patients with CF diagnosed after false-negative screening results, and ratio of CF to cases with positive cystic fibrosis screening and inconclusive diagnosis (CFSPID).

**Results:**

The DGNS has 767 datasets of newborns with CF/CFSPID and the DMR has 910 confirmed cases of CF/CFSPID. A false-negative screening was reported by the DGNS for 37/767 (4.8%) and by the DMR for 49/910 (5.4%). The ratio of CF to CFSPID is 17.4:1 (DGNS, 2017–2020) and 28.1:1 (DMR), respectively.

**Discussion:**

The DGNS and the DMR, each with different strengths in the documentation of screening (DGNS) and diagnostic data (DMR), provide important clues for the number of newly diagnosed cystic fibrosis patients after the introduction of NGS. Legal requirements for tracking those screened, recording all children with CF, and exchanging data between the DGNS and DMR could improve evaluation in the future.

## Hintergrund

Die Mukoviszidose, auch zystische Fibrose (engl. Cystic Fibrosis – CF) genannt, ist die häufigste lebensverkürzende autosomal-rezessiv vererbte Multisystemerkrankung in Deutschland [1] mit einer Inzidenz von 1:4754 [2] bis 1:5187 (4421–6086; [3]) Neugeborenen. Ursächlich für die Mukoviszidose ist eine Funktionsstörung des sogenannten Cystic-Fibrosis-transmembrane-regulator-(CFTR-)Proteins, das als Kanal für die Leitung von negativ geladenen Ionen wie Chlorid durch die Zellmembran fungiert. Im letzten Jahrzehnt wurden Modulatoren der CFTR-Funktion entwickelt, die bei einem Großteil der Betroffenen die Funktion zumindest teilweise wiederherstellen können und damit Lebenserwartung, Lebensqualität und Gesundheitszustand der Menschen mit Mukoviszidose erheblich verbessern [4].

Zum 01.09.2016 wurde vom Gemeinsamen Bundesausschuss (G-BA) das generelle Neugeborenenscreening auf Mukoviszidose in Deutschland als spezielle Früherkennungsuntersuchung der „Kinder-Richtlinie“ eingeführt. Das Screening auf Mukoviszidose wird 3‑stufig als serielle Kombination von 2 biochemischen Tests auf immunreaktives Trypsin (IRT) und Pankreatitis-assoziiertes Protein (PAP) sowie einer DNA-Mutationsanalyse auf die 31 häufigsten CFTR-Mutationen durchgeführt. Bei Nachweis mindestens einer Mutation oder eines IRT-Werts oberhalb der 99,9. Perzentile (Fail-Safe, Safety Net)^1^ gilt das Screening als auffällig (positiv). Das Labor teilt einen positiven Screeningbefund zusammen mit einer Liste möglicher Einrichtungen für die Konfirmationsdiagnostik dem Einsender des Screenings mit, der dann wiederum die Eltern informiert [5]. Dem Beschluss waren jahrelange Diskussionen über die Ziele des Screenings und den Stellenwert der genetischen Untersuchung vorausgegangen. Mit der Entscheidung gegen ein IRT-DNA-Verfahren und für ein IRT-PAP-DNA-Verfahren mit Fail-Safe wurde die Anzahl der genetischen Untersuchungen reduziert, das positive Screeningergebnis vom Nachweis eines Carrier-Überträgerstatus entkoppelt [5] und es wurden mehr falsch-positive Screeningergebnisse (gegenüber anderen international eingesetzten Screeningverfahren) in Kauf genommen [2, 3].

Auf ein Tracking, d. h. die Sicherstellung, dass alle auffälligen Screeningbefunde weiter abgeklärt werden, wurde u. a. mit Hinblick auf den Datenschutz in Deutschland verzichtet und die Nachverfolgung der Konfirmationsdiagnostik im Sinne einer Qualitätssicherungsmaßnahme in die Hände der durchführenden Screeninglabore/-zentren gelegt [5]. Die Screeninglabore/-zentren sind dabei auf die freiwillige Rückmeldung der Ergebnisse der Konfirmationsdiagnostik durch die Einrichtungen angewiesen, die die Konfirmationsdiagnostik durchführen. Die Deutsche Gesellschaft für Neugeborenenscreening (DGNS) erstellt seit 2006 jährlich zur Erfassung der in der Kinder-Richtlinie definierten Qualitätsparameter auf Basis der Daten aller 11 deutschen Screeninglabore einen Screeningreport mit kumulativen Screeningparametern und der Konfirmationsdiagnostik bei bestätigten Fällen, u. a. auch Daten zur Mukoviszidose [6].

Mit dem Deutschen Mukoviszidoseregister (DMR) steht seit 1995 ein freiwilliges Patientenregister für teilnehmende Mukoviszidosebehandler zur Verfügung. Hier werden die medizinischen Daten der Menschen mit gesicherter Diagnose „Mukoviszidose“ dokumentiert, wenn diese in das Verfahren eingewilligt haben. Ziel des DMR ist die strukturierte Erfassung und Auswertung der Daten von Menschen mit Mukoviszidose in Deutschland zur Beurteilung und Optimierung der Versorgungsqualität in den teilnehmenden Mukoviszidose-Einrichtungen. [7]. Bei der Auswahl des genetischen Untersuchungsspektrums des Neugeborenenscreenings wurde auf die epidemiologischen Daten aus dem DMR zurückgegriffen und die häufigsten 31 in Deutschland berichteten krankheitsverursachenden CFTR-Mutationen ausgewählt [5].

Aus dem Neugeborenenscreening kann sich die Konstellation eines positiven Mukoviszidosescreenings und einer unklaren Konfirmationsdiagnostik (engl. „CF-screening positive, inconclusive diagnosis” – CFSPID) ergeben [8]. In Abhängigkeit vom Einsatz eines erweiterten genetischen Untersuchungsspektrums liegt das Verhältnis CF:CFSPID zwischen 1,3:1 (Polen) und 31:1 (Niederlande; [3]). Das Ziel des Screenings in Deutschland ist die Erkennung von Menschen mit Mukoviszidose zur unverzüglichen Therapieeinleitung im Krankheitsfall und nicht die Erkennung von Menschen mit CFSPID [5].

In Ermangelung eines epidemiologischen Goldstandards zur Ermittlung der Anzahl von diagnostizierten Neugeborenen mit Mukoviszidose und CFSPID ist das Ziel dieser Arbeit, die Daten der DGNS zu den in der Konfirmationsdiagnostik bestätigten Fällen und die vom DMR erfassten Diagnosehäufigkeiten für Kinder, die zwischen dem 01.01.2017 und dem 31.12.2021 geboren sind, zu vergleichen und die Limitationen und Stärken beider Datenquellen darzustellen.

## Methoden

Der DGNS werden im Rahmen der Qualitätssicherung von allen deutschen Screeninglaboren/-zentren mit einer Accessdatenbank Daten zum Neugeborenenscreening übermittelt. Das Verfahren orientiert sich an den in der Kinder-Richtlinie definierten Qualitätskriterien. Nach der Richtlinie soll das Ergebnis der Konfirmationsdiagnostik zur Qualitätssicherung des Screenings von den Einrichtungen an das zuständige Screeninglabor/-zentrum übermittelt werden. Weil ein offizielles Tracking fehlt, erfolgen diese Rückmeldungen auf freiwilliger Basis. Einige Screeninglabore/‑zentren führen eine aktive Nachfrage in den von ihnen empfohlenen Konfirmationszentren durch. Die DGNS und die Screeningzentren stellen für die Rückmeldung der Konfirmationsdiagnostik einen strukturierten Fragebogen [9] zur Verfügung, der folgende Daten erfasst:

- Screening-ID,
- Stammdaten (Geburtsdatum, Geschlecht, ethnische Zugehörigkeit),
- Diagnose,
- Diagnosedaten (klinische Hinweise, pränatale Diagnose, Familienanamnese, Mekoniumileus, sonstige),
- Durchführung und Ergebnis des Schweißtestes,
- Ergebnis der genetischen Untersuchung (in der Regel keine Übermittlung der genetischen Varianten) und
- Angaben zur Geburt und bei Vorstellung: Schwangerschaftsdauer sowie Gewicht und Länge des Neugeborenen.

Die DGNS veröffentlicht, wie bereits erklärt, jährlich den Screeningreport [6]. Hierfür werden nur für die in der Konfirmationsdiagnostik bestätigten Fälle die pseudonymisierten Angaben des Erhebungsbogens zusammen mit den im Screeninglabor vorliegenden Daten der Screeningparameter (Screeningzeitpunkt, Werte für IRT, ggf. PAP, Mutationsanalyse) erhoben, auf Doppelmeldungen geprüft und extern validiert. Im Rahmen der Validation wird auch die vom Labor angegebene Diagnose CF oder CFSPID überprüft. Unauffällige oder offene Fälle werden nicht an die DGNS übermittelt.

Das Deutsche Mukoviszidoseregister (DMR) wird vom Mukoviszidose e. V. seit 1995 betrieben und ist ein freiwilliges Patientenregister auf der Grundlage eines Registerprotokolls und eines Datenschutzkonzeptes (Ethikvotum Justus-Liebig-Universität Gießen, FB Medizin, AZ 24/19). Nach Aufklärung und Einholung eines Einverständnisses der Sorgeberechtigten und nach Bestätigung einer der folgenden Diagnosen (CF, CFSPID oder CFTR-assoziierte Erkrankung) melden die am DMR teilnehmenden Mukoviszidoseeinrichtungen in Deutschland (2021: 87 Einrichtungen) folgende Daten:

- Stammdaten (Geburtsdatum, Geschlecht, Geburtsort, ethnische Zugehörigkeit),
- Diagnosedaten (klinische Hinweise, Ergebnis des Schweißtestes, Ergebnis der genetischen Untersuchung, einschl. Varianten, „Neugeborenenscreening durchgeführt“ und qualitatives Ergebnis des Screenings) und
- klinische Verlaufsdaten.

Dies schließt alle Menschen mit CF ein, die in Deutschland behandelt werden unabhängig von ihrem Geburtsland. Seit 2015 werden neben der Diagnose Mukoviszidose auch die Diagnosen CFSPID und CFTR-assoziierte Erkrankungen erfasst. Zur Vermeidung von Dubletten erfolgt eine zentrale Pseudonymisierung. Die Daten werden webbasiert erfasst und mittels aktiven Datenbankmanagements durch das Interdisziplinäre Zentrum für Klinische Studien der Universitätsmedizin Mainz qualitätskontrolliert. Jährlich werden Berichtsbände veröffentlicht und die Daten für Forschungsanfragen in aggregierter und anonymisierter Form zur Verfügung gestellt [7].

Für die Auswertungen werden deskriptiv die Diagnosedaten der 2017–2021 geborenen Neugeborenen der DGNS (Datenstand 14.04.2023) und des DMR (Datenstand 12.04.2023) dargestellt und bezüglich der Diagnosehäufigkeit und der Rate falsch-negativ Gescreenter verglichen. Für die Auswertungen des DMR werden CFTR-assoziierte Erkrankungen mit CFSPID zusammengefasst, weil deren diagnostische Abgrenzung im Neugeborenenalter schwierig ist.

## Ergebnisse

Für die zwischen 2017 und 2021 Geborenen liegen der DGNS insgesamt 767 Datensätze von Fällen mit bestätigter Mukoviszidose bzw. CFSPID vor. Für 2017–2020 wurden dabei 563 CF- und 32 CFSPID-Fälle gemeldet sowie 13 Fälle, bei denen die Angaben nicht für eine Unterscheidung zwischen CF/CFSPID ausreichten. Die 159 im Jahr 2021 geborenen CF-/CFSPID-Fälle sind noch nicht endgültig validiert. Vom DMR werden 910 (879 CF, 31 CFSPID) Datensätze von Menschen mit einer bestätigten Diagnose berichtet (Tab. 1). Die Anzahl der diagnostizierten Fälle liegt in den jeweiligen Geburtsjahren zwischen 145 und 161 (DGNS) bzw. zwischen 151 und 213 (DMR). Die Rate von CF:CFSPID beträgt nach DGNS 17,6:1 (2017–2020) nach DMR 28,4:1 (2017–2021).

**Table 1:** 

	DGNS 2017–2020	DGNS 2021	DMR 2017–2021
CF	563	–	879
CFSPID	32	–	31
CF/CFSPID?	13	159*	–
*Summe CF und CFSPID*	*608*	*159*	*910*
Ratio CF:CFSPID	17,6:1	–	28,4:1

* Noch nicht validiert

Die Diagnose wurde teilweise ohne Durchführung eines CF-Screenings dokumentiert (DMR: 66 Fälle, DGNS: 1 Fall). Im DMR fehlen bei 35 Patienten die Angaben zur Durchführung eines Screenings, in den DGNS-Daten sind die Screeningergebnisse vollständig vorhanden. In den DGNS-Daten wurden bis 2018 nur die bestätigten Fälle erfasst. Erst seit 2019 wird zusätzlich abgefragt, ob auffällige Screeningbefunde in der Konfirmation unauffällig waren oder nicht abgeklärt wurden. In den Daten von 2019 wurden von den Laboren (*n* = 8), die diese neu geforderten Angaben schon aus ihrer Datenbank auslesen konnten, 475 auffällige CF-Screeningbefunde gemeldet, von denen in der Konfirmationsdiagnostik 98 bestätigt wurden, 348 unauffällig waren und 29 (6,1 %) nicht abgeklärt wurden („lost to follow-up“).

In den Daten der DGNS wurde bei 37 der 767 Neugeborenen mit CF/CFSPID (4,8 %) die Diagnose nach einem falsch-negativen Screening aufgrund klinischer Hinweise eines Mekoniumileus (MI^2^; 14 ×), einer positiven Familienanamnese (4 ×) oder klinischer Symptome (11 ×) gestellt. Grund für das falsch-negative Screeningergebnis war in 18 Fällen ein unauffälliger IRT-Wert (9 davon bei MI), in 15 Fällen ein unauffälliger PAP-Wert (4 bei MI) und bei 4 Neugeborenen war im Screeningalgorithmus keine der 31 Mutationen nachweisbar. In den Daten der DMR wird für 49 der 910 (5,4 %) Menschen mit CF/CFSPID resp. 49 der 879 Menschen mit CF (5,6 %) im DMR ein falsch-negatives Screening berichtet. Im DMR lagen Mekoniumileus (8 ×), klinische Symptome (28 ×), positive Familienanamnese (2 ×) als klinische Hinweise auf eine Mukoviszidose vor. Für 8 (DGNS) bzw. 11 (DMR) Menschen mit CF/CFSPID wurden keine Angaben zu den klinischen Hinweisen, die zur Diagnose führten, gemacht. Bei 44 von 49 falsch-negativ gescreenten Menschen mit CF (90 %; DMR) bzw. 26 von 31 CF/CFSPID (84 %; DGNS; 6 × keine Angaben zur Genetik bekannt) war in der Genetik mindestens eine der Mutationen aus dem Panel der 31 CFTR-Mutationen des Screeningalgorithmus nachweisbar.

## Diskussion

Von den zwischen 2017 und 2021 geborenen Kindern wurden 767 (DGNS) bzw. 910 (DMR) mit CF/CFSPID in den Datenbanken erfasst. Grund für die Differenz ist, dass in den DGNS-Daten zwar praktisch alle auffälligen Screeningergebnisse erfasst werden, aber die Rückmeldung der Konfirmationsdiagnostik an die Labore nicht verpflichtend ist (2019: Lost-to-follow-up-Rate: 6,8 %). Außerdem werden die später diagnostizierten Kinder ohne oder nach unauffälligem CF-Screening nicht systematisch an die Labore gemeldet und können daher nicht in den DGNS-Daten berücksichtigt werden. Zusätzlich werden in das DMR auch Daten von in Deutschland behandelten, aber nicht in Deutschland geborenen Menschen mit Mukoviszidose aufgenommen.

Das Neugeborenenscreening auf Mukoviszidose weist im Vergleich zu anderen Zielkrankheiten des Erweiterten Neugeborenen-Screenings eine relativ geringe Spezifität und Sensitivität auf. Falsch-negative Screeningergebnisse sind daher ein zu erwartendes Ereignis. Die Rate von 5 % nach einer Nachbeobachtungszeit von max. 6,5 Jahren bestätigt die ursprünglichen Voraussagen der Sensitivität von etwa 94 % des CF-Screeningalgorithmus [5]. Dabei ist bei der Mukoviszidose auch zu berücksichtigen, dass 15 % der Betroffenen bei Geburt einen Mekoniumileus aufweisen und bei dieser Konstellation bis zu 18 % der falsch-negativen Screeningergebnisse berichtet werden [10].

Ziel des Screenings ist die Früherkennung der Mukoviszidose und nicht die Detektion von Neugeborenen mit positivem Screeningergebnis und unklarer Konfirmationsdiagnostik (CFSPID; [5]), auch wenn bei einem Teil dieser Kinder (10–20 %) im Verlauf eine Mukoviszidose aufgrund klinischer Hinweise oder einer im Verlauf geänderten Beurteilung der Konfirmationsdiagnostik berichtet wird [6, 11]. Das DMR zeigt mit einem Verhältnis von 28,4 CF:1 CFSPID eine im internationalen Vergleich [3] geringe Rate von CFSPID-Patienten auf; in den DGNS-Daten ist der Anteil der CFSPID-Diagnosen mit einem Verhältnis von 17,6:1 niedriger. Dies könnte damit begründet sein, dass CFSPID-Fälle bei fehlenden Angaben zur Genetik ausschließlich aufgrund der intermediären Chloridwerte des Schweißtests definiert wurden, obwohl eigentlich auf Basis der Genetik die Definition CF oder Ausschluss einer Mukoviszidose korrekt gewesen wäre.

Eine wichtige und populationsspezifische Komponente des Screeningverfahrens ist das genetische Panel. Die Auswahl der 31 häufigsten krankheitsverursachenden Mutationen für das deutsche Screening hätte bei 90 % der falsch-negativ gescreenten Neugeborenen mindestens 1 Variante (DMR) erfasst. Eine Erweiterung des genetischen Untersuchungspanels im Sinne eines Next-Generation Sequencing (NGS) würde die Anzahl unklarer Konfirmationsdiagnostik aufgrund des erhöhten Nachweises von Varianten unklarer Krankheitsrelevanz erhöhen, wie dies u. a. in Polen der Fall ist [11].

Die beiden Datenquellen können den bewussten Verzicht des G‑BA auf eine gesetzlich festgelegte Pflicht zur Meldung der Konfirmationsdiagnostik an die Screeninglabore (Tracking) nicht ersetzen, aber trotz Limitationen die Evaluation des CF-Screenings in Deutschland unterstützen. Das DMR umfasst die Daten von Mukoviszidosebehandlern aus 87 Zentren. Seine Repräsentativität ist aufgrund der fehlenden gesetzlichen Dokumentationspflicht limitiert, da nicht alle Behandler in Deutschland teilnehmen, ein Einverständnis der Sorgeberechtigten zur Übermittlung der Daten an das Register Voraussetzung ist und im DMR auch Menschen erfasst werden, die nicht in Deutschland geboren sind. Die Repräsentativität liegt – in Ermangelung von offiziellen Diagnosezahlen – nach eigenen Berechnungen des DMR bei über 90 % für Kinder und Jugendliche mit Mukoviszidose; für Menschen mit CFSPID können keine entsprechenden Angaben gemacht werden. Die Rate nicht einwilligender Sorgeberechtigter liegt nach eigenen Schätzungen des DMR bei < 5 %. Der DGNS-Screeningreport erfasst alle Parameter des CF-Screenings, setzt aber aufgrund der fehlenden datenschutzrechtlichen Grundlage und gesetzlichen Verpflichtung eine freiwillige, aktive Meldung der Konfirmationsdiagnostik durch den Behandler voraus. Eine Erhebung 2019 zeigte, dass über alle 13 Screeninglabore/-zentren in 300 von 799 Fällen (38 %) das Ergebnis der Konfirmationsdiagnostik unklar blieb („lost-to-follow-up“ oder „lost to documentation“), da unauffällige Befunde bisher nicht systematisch in den DGNS-Daten erfasst wurden [3]. Für Labore, die unauffällige Fälle an die DGNS melden können, lag die Lost-to-follow-up-Rate 2019 bei 6,8 %. Ein systematisches, aktives Tracking senkt bei vertretbaren Kosten die Lost-to-follow-up-Quote auf 1 % [12] und erhöht die Zufriedenheit der Eltern [13].

Das Screening auf Mukoviszidose hat nach den Daten des DMR zu einer früheren Diagnosestellung beigetragen: 2021 waren die Kinder im Median 0,08 Jahre alt (vgl. 2015: 0,92; [4]). Anhand der Daten von DGNS und DMR lässt sich – trotz ihrer Limitationen – erkennen, dass das Mukoviszidosescreening mit einer Rate falsch-negativer Screeningergebnisse von 5 %, einer hohen Erfassung genetischer Varianten mit dem ausgewählten Panel und der geringen Quote von CFSPID (28,4:1) die gesetzten Vorgaben erreicht hat. Die bewusst in Kauf genommene geringere diagnostische Güte des Mukoviszidosescreenings im Vergleich zu anderen Zielerkrankungen des erweiterten Neugeborenenscreenings – mit nur einer bestätigten Mukoviszidosediagnose bei 5 auffälligen Screeningbefunden – ist bei der Reevaluierung des Screeningalgorithmus durch den G‑BA zu diskutieren.

Die Stärken der DGNS-Qualitätssicherung liegen in der vollständigen Erfassung der Screeningdaten mit Darstellung der Gründe bei falsch-negativem Screeningbefund und in der Lost-to-follow-up- oder Lost-to-documentation-Rate nach einem auffälligen Screeningbefund. Die Stärken des DMR liegen hingegen in der vollständigeren Diagnoseerfassung und dem dokumentierten Langzeitverlauf der Menschen mit Mukoviszidose. Der Verzicht auf ein verpflichtendes Tracking, eine verpflichtende Dokumentation von Menschen mit Mukoviszidose in einem Patientenregister und die fehlende Verknüpfung der Datenquellen DGNS und DMR behindern aktuell die Evaluation, die Optimierung des Screeningverfahrens und die Beurteilung des mittel- und langfristigen Nutzens des Neugeborenenscreenings auf Mukoviszidose in Deutschland. Es ist daher dringend geboten, diese aus Sicht aller Beteiligten schmerzhaften Lücken zwischen Erfassung des Screenings (DGNS) und Diagnose und Verlauf (DMR) zu schließen, um das Ziel des Screenings – die unverzügliche Therapieeinleitung im Krankheitsfall– für alle Betroffenen zu erreichen und das Screening von Anfang bis Ende zu evaluieren.

## Footnotes

1 Fail-Safe oder Safety Net („Sicherheitsnetz“) soll eine zusätzliche Sicherheit im Screeningalgorithmus bewirken, damit Kinder mit seltenen CFTR-Mutationen nicht übersehen werden.

2 Darmverschluss (Ileus) des Neugeborenen durch Kindspech (Mekonium).
